# Fuzzy association rules for biological data analysis: A case study on yeast

**DOI:** 10.1186/1471-2105-9-107

**Published:** 2008-02-19

**Authors:** Francisco J Lopez, Armando Blanco, Fernando Garcia, Carlos Cano, Antonio Marin

**Affiliations:** 1 Department of Computer Science and AI, University of Granada, 18071, Granada, Spain; 2 Department of Genetics, University of Seville, 41012, Seville, Spain

## Abstract

**Background:**

Last years' mapping of diverse genomes has generated huge amounts of biological data which are currently dispersed through many databases. Integration of the information available in the various databases is required to unveil possible associations relating already known data. Biological data are often imprecise and noisy. Fuzzy set theory is specially suitable to model imprecise data while association rules are very appropriate to integrate heterogeneous data.

**Results:**

In this work we propose a novel fuzzy methodology based on a fuzzy association rule mining method for biological knowledge extraction. We apply this methodology over a yeast genome dataset containing heterogeneous information regarding structural and functional genome features. A number of association rules have been found, many of them agreeing with previous research in the area. In addition, a comparison between crisp and fuzzy results proves the fuzzy associations to be more reliable than crisp ones.

**Conclusion:**

An integrative approach as the one carried out in this work can unveil significant knowledge which is currently hidden and dispersed through the existing biological databases. It is shown that fuzzy association rules can model this knowledge in an intuitive way by using linguistic labels and few easy-understandable parameters.

## Background

The availability of the complete genome from diverse species and the advent of high throughput genomic technologies, have generated a great amount of structural and functional information boosting Bioinformatics research to develop computational techniques that help to analyze such a huge amount of data [1]. Many computer science techniques have been applied over biological data [2,3]. More particularly, in the gene expression data analysis field, Eisen et al. [4] applied hierarchical clustering to identify functional groups of genes. Tamayo et al. developed the package GENECLUSTER [5], which makes use of the self-organized maps to extract gene expression patterns. To address some problems that present the classical clustering algorithms Hastie et al. [6] proposed the *Gene Shaving *algorithm. For a review on cluster algorithms for gene expression analysis see [7]. Association rules have also been previously used in Bioinformatics. For example, Rodriguez et al. [8] used a modified version of the *Apriori *algorithm to get relations between protein sequences and protein features, and more recently, Hermert et al. [9] and Dafas et al. [10] used association rules for analyzing gene expression data.

Nevertheless, most of these works focus on the analysis of a single-source dataset (e.g. a gene expression matrix). The Bioinformatic community has recently realized about the importance of the integration of information obtained from diverse sources in order to place the data into an useful context, obtaining as much knowledge as possible from their analysis [11-14]. Another key point is the heterogeneity of biological data, i.e. these data can be found in the form of ontologies, sequences, measures etc. Although some approaches that carry out analysis of heterogeneous information are emerging, there is still a lack of integrative approaches able to handle a broad variety of types of data. In addition, biological data is known to be imprecise and noisy. Classical crisp techniques as the ones reported above are usually applied to analyze biological data. However, other methods which are known to perform better when dealing with imprecise and noisy data (e.g. fuzzy techniques) are barely used.

Traditional statistical techniques are also typically used to analyze biological data. For example, Marin et al. [15] studied relationships between the gene expression level and the G+C content of the gene, showing that the amount of mRNA transcripts of genes with a high G+C content is higher than the amount of mRNA transcripts of those with a lower G+C content. In this work they also studied the negative correlation between the gene length and its G+C content. Other relations between the amount of specific mRNA and gene sequence features have also been studied by Coghlan & Wolfe [16] and Jansen & Gerstein [17]. However, the nature of statistical techniques makes hard the integration of diverse heterogeneous data into the analysis. Furthermore, these works focus only on the study of few potential relations between the biological variables they consider.

We here conduct a fuzzy-integrative approach merging genomic information from different sources and of various types by using the well-known association rule mining techniques. The primary goal of this paper is to present a novel fuzzy association rule extraction method based on the Top-Down Frequent Parent Growth (TD-FP Growth) algorithm [18] to find relationships between a diversity of genomic characteristic comprising both structural and functional features. Fuzzy set theory is specially suitable to model imprecise data while association rules are very appropriate to carry out an integrative analysis of heterogeneous data, thus a fuzzy association rule mining algorithm is a suitable method for our purposes. Furthermore, unlike previous works such as those cited above which studied few potential relations between structural and functional genomic features [15,19,20], our approach allows to examine all the existing associations between very different features (e.g. expression levels, Gene Ontology annotations, gene length, G+C content etc.).

In 1993, Agrawal proposed an algorithm for extracting association rules from large databases [21]. Since then, association rule mining has become one of the main techniques for Knowledge Discovery in Databases (KDD). Given a transactional database, where each transaction is a set of attribute-value pairs or *items*, the aim of these techniques is to find a set of expressions of the form *X *→ *Y*, where *X *and *Y *are sets of attribute-value pairs or *itemsets*. This expression is called *association rule*, and indicates that if *X *occurs then *Y *is likely to occur. The probability that *Y *occurs, given that *X *has occurred, is called the *confidence *of the rule. The probability that both *X *and *Y *occur is called the *support *of the rule. Thus, classical association rule mining algorithms aim to extract association rules with support and confidence greater than some user-specified threshold.

Fuzzy set theory was proposed by Zadeh in 1965 to mathematically model the imprecision inherent to some concepts [22]. Briefly, fuzzy set theory allows an object to partially belong to a set with a membership degree between 0 and 1. Likewise, fuzzy logic allows a statement to be true with a certainty degree between 0 and 1. Classical set theory and logic are special cases of their fuzzy counterparts in which membership and certainty degrees are restricted to be either 0 or 1. Fuzzy concepts have been successfully applied to many different areas, including control, pattern recognition, and data mining (e.g. classification and clustering) [23].

Association rule mining often needs to deal with imprecise or uncertain concepts. In this particular case, some concepts (i.e. linguistic labels) need to be defined over continuous attribute domains. Classical quantitative association rule mining methods partition these continuous domains into crisp intervals. Fuzzy logic is proved to be a superior technology to enhance the interpretability of these intervals [24]. The *fuzzification *of the continuous domains is carried out by partitioning them into fuzzy sets. Fuzzy confidence and support measure the significance of the rule. Thus, fuzzy association rules are expressions of the form *X *→ *Y*, but in this case, *X *and *Y *are sets of fuzzy attribute-value pairs. In addition, in order to avoid some of the drawbacks of the classical confidence/support framework, *Certainty Factors *(CFs) were also used in our analysis to measure the quality of the rules [25].

We have chosen the yeast *S. cerevisiae *genome as a benchmark, since intensive work on this model organism has provided high quality datasets and also abundant literature exploring the trends and patterns in genomic organization and function. The yeast *Saccharomyces cerevisiae *was the first eukaryote to have its genome sequenced [26]. Since then, work with this organism has led the way in structural and functional genomics, setting the standard for the global analysis of cellular and molecular biology and paving the way for similar approaches in other organisms [19,27,28].

Most of the huge amount of biological information about *S. cerevisiae *is stored in databases such as the Saccharomyces Genome Database (SGD [29,30]), the Comprehensive Yeast Genome Database (CYGD [31]) and others. Recent information resulting from forefront biological research not yet included in the databases has to be compiled from the pertinent literature.

The fuzzy association rule mining method has been run over a yeast data table containing information about the size and base composition of genes and upstream intergenic sequences, transcriptional orientation, presence of TATA box, gene's protein amount produced during normal growth, gene responsiveness to changing conditions, Gene Ontology labels and gene expression changes obtained from the datasets by Cho et al. [32] and Gasch et al. [33]. Among the rules extracted we have found most of the previously reported trends relating these variables, but also some new associations which may contribute to the framing of genomic structural and functional relationships.

## Results and discussion

### Dataset

The following subsections describe the information included in the data table, where rows correspond to genes and columns to the different gene features included in the analysis.

#### Structural features

The yeast genome sequence and annotation was downloaded from the SGD ftp server (release of February 2007). The following genomic structural variables were included in the data table:

• Gene length: the number of nucleotides in the coding sequence.

• Gene G+C content: the proportion of guanine plus cytosine.

• Intergenic length: the number of nucleotides in the gene upstream sequence.

• Intergenic G+C content: the proportion of guanine plus cytosine in the intergenic sequence.

• Gene Orientation: *divergent*, if the promoter region of the gene is beside the promoter region of another gene, or *tandem*, if the promoter region of the gene is beside the end of another gene.

#### Functional features

Regarding the activity of a gene, two key functional features are the amount of its final product and its ability to change the expression level in response to changing conditions. The first magnitude has been measured by Ghaemmaghami et al. [34] providing a precise estimation of the number of protein molecules per cell for 75% of the yeast genes during normal growth. The second feature has been measured by Tirosh et al. [35] by using a data set of yeast expression profiles for more than 1500 conditions to calculate the response of each gene to changing conditions. Each gene was assigned a Responsiveness measure based on the variability of its expression pattern, defined as the sum of squares of the log2-ratios over all conditions. A third variable, related to gene expression level and responsiveness, is the presence (absence) of a TATA box, a conserved element element of the promoter that functions in the transcription initiation. Tirosh et al. [35] reported the presence of a TATA box in 585 yeast genes and its absence in 2492 genes, and noted that TATA boxes tend to occur more frequently in genes of particular functions.

**Figure 1:**
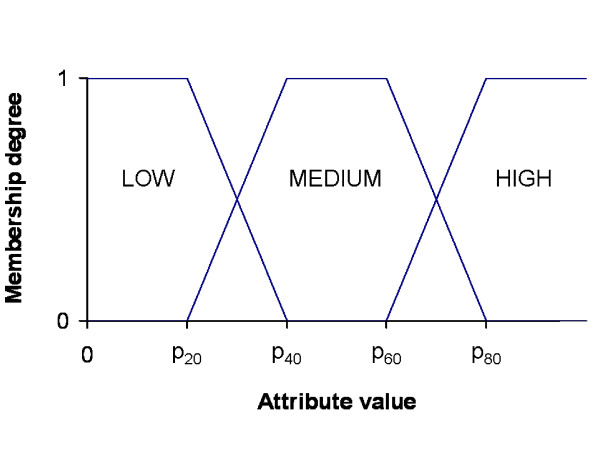
**Linguistic labels defined for continuous features**. This figure describes how the membership functions are defined for each fuzzy set in the corresponding continuous domain.

Note that the domains of some of these attributes and of the attributes in the previous paragraph are continuous (e.g. Responsiveness, Protein abundance, Length etc.). These domains are partitioned into three fuzzy sets which represent the linguistic labels HIGH, MEDIUM and LOW. Fuzzy sets are defined by using the expert-guided percentiles *p*_20_, *p*_40_, *p*_60_, *p*_80 _as shown in Figure 1. The experiments were also run defining more fuzzy sets on the domains (4, 5 and 6). However, the obtained results did not improve the rule sets obtained with three fuzzy sets (results not shown). In addition, since the use of only three linguistic labels provides clearer rule sets and improves the performance of the methodology (less itemsets), we decided that it was not needed a greater granularity in the analysis and therefore only these fuzzy sets were defined. More granularity can be easily obtained in the analysis by defining more fuzzy sets in case it is needed.

#### Gene Ontology annotations

The Gene Ontology (GO) Consortium has become *de facto *a standard for describing gene products in databases. It provides a structured, controlled vocabulary for describing the roles of genes and gene products in any organism. GO consists of three separate ontologies: Biological process describes to what biological objectives genes and gene products can contribute, molecular function describes their biochemical activity, and cellular component refers to the place in the cell where they can be located. The terms (nodes) in the GO database form a Directed Acyclic Graph (DAG), in which terms are children of one or several more general terms. This means that the closer a term is to the root, the more general it is, and the closer a term is to the leaf, the more specific it is. Genes and gene products are annotated in one or more terms by collaborating databases at the most specific level possible, but are considered to share the attributes of all the parent nodes.

Two main strategies can be followed when including GO annotations into the final data table:

• Select a level of the DAG, and include only terms of this level in which genes are explicitly or implicitly annotated. However, GO levels are not homogeneous, i.e. terms representing general concepts and others which represent more specific concepts are found in the same GO level. Therefore, some information might be lost when using this strategy.

• Consider all the terms in which genes are explicitly or implicitly annotated. The *information content *(IC) of each node is calculated to discriminate useful terms [36]. Finally, insert into the table those terms for which the information content is over a user-specified threshold. The information content of a node is computed as follows:

$$IC(T)=\frac{-\log P(T)}{-\log P(min)},$$

where *P*(*T*) represents the probability of finding T or a child of T in the ontology. The denominator is used to normalize, i.e *P*(*min*) = 1/*TotalNumberOfAnnotations*.

Since we wanted to avoid any previous loss of information we selected the second strategy for the construction of our data table. GO terms for each gene were obtained from the Gene Ontology webpage [37] (release of January 2007).

#### Microarray data

Microarrays allow to measure expression of thousands of genes simultaneously. This technology has become the source of large volumes of data organized in the so-called gene expression matrices. The analysis of these matrices allows to get information about cellular operation in organisms. However, this analysis is very complex due to: *i*) the large number of genes (even in the simplest organisms), *ii*) the unlimited number of conditions under the genes can be studied, and *iii*) the noise that affects the whole process [38].

The microarray data analyzed here are those obtained by Cho et al. [32] and Gasch et al. [33]. The first expression dataset contains the expression levels of 2879 yeast genes under 17 cell cycle conditions that cover approximately two full cell cycles. The dataset by Gasch et al. contains the expression levels of 6152 genes under 172 experimental conditions.

Most of previous works which use association rules for analyzing microarray data map real gene expression values to crisp labels [14,39]. Some problems arise when following this strategy: some thresholds must be selected that determine whether genes are expressed or not. In addition, it should also be considered the problem of the loss of information associated to every discretization process. But probably the most important drawback of this strategy is its necessity to include one column into the data table for each microarray condition (i.e. 17 and 172 columns). This means that a huge number of itemsets and rules involving gene expression levels are generated. Thus, the interpretation of the resultant rule set is very difficult. Furthermore, it is hard to identify gene expression profiles and to relate them to structural or to other functional features.

In a previous work [40], we explore the use of clustering algorithms to obtain groups of genes with similar expression profiles. Hence, only one column indicating the cluster(s) to which every gene belongs has to be introduced into the data table, thus avoiding the huge number of rules involving gene expression levels. However clustering shows the limitation that genes are grouped according to their behaviour under all conditions. In this work we propose a strategy based on the well known *biclustering techniques *[41]. By running a biclustering algorithm over the gene expression matrix we obtain groups (biclusters) of genes which behave similarly under certain conditions (not necessarily all of them).

Different biclustering algorithms may provide different bicluster sets [42,43]. Although many of the biclusters obtained by two different algorithms probably overlap, slight differences may be found between these overlapping biclusters and also, we might find biclusters with one of the algorithms that are not generated by the other and vice versa. In order to provide a better and broader coverage of the existing gene expression profiles we ran two different biclustering algorithms. The two selected methods are *Gene-&-Sample shaving *and *EDA *biclustering algorithms, which performance has been proved (work currently submitted). The former uses Principal Component Computation to identify biclusters, extending the Gene Shaving algorithm proposed by *Hastie et. al*. [6]. The latter makes use of a particular type of Evolutionary Algorithms called Estimation of Distribution Algorithms (EDAs) to identify biclusters in gene expression matrices. Both algorithms look for high between-sample variance biclusters. Thus, their results reveal genes with very different behavior across samples (genes involved in constantly activated processes as well as those involved in none of the active processes are ignored), so they become very useful for identifying distinct types of samples and the features which may produce these differences.

### Experiments

Certainty Factors (CFs), Confidence and Support thresholds were varied in each study to obtain a number of rules which could be easily analyzed by the expert. The threshold values and the total number of rules obtained in each experiment are detailed in Table 1. When selecting the quality thresholds one must take into account the type of data that are being analyzed. For example, if the support of an itemset is very high, it very likely appears in the consequent of many associations which are obtained by chance, since it appears in many transactions of the data table [25]. Therefore, low values for CF are expected (e.g. rules in Table 2). Some previous knowledge about the data distribution is needed in order to determine what should be considered acceptable and, according to this, modify the quality thresholds. However, in most cases this is not an issue since the data distribution is known. For example, in our case we know that the support of *orientation *= *DIV ERGENT *and *orientation *= *TANDEM *is approximately 0.5, and that the support of the items involving GO terms are quite low (usually under 0.01).

**Table 1:** Thresholds and total number of rules

Variables	CF & Conf. threshold	Support threshold	Total number of rules	FDR
Structural variables	0.1	0.01	24	0.093
Molecular Function & Structural variables	0.4	0.004	20	0.042
Biological Process & Structural variables	0.5	0.004	7	0.050
Cellular Component & Structural variables	0.5	0.004	12	0.011
Protein abundance & Responsiveness & TATA box	0.1	0.002	15	0.000
Protein abundance & Structural variables	0.1	0.002	4	0.040
Protein abundance & Molecular Function	0.2	0.002	19	0.109
Protein abundance & Biological Process	0.4	0.002	21	0.005
Protein abundance & Cellular Component	0.3	0.002	14	0.011
Responsiveness & Structural variables	0.1	0.002	10	0.044
Responsiveness & Molecular Function	0.3	0.002	23	0.069
Responsiveness & Biological Process	0.6	0.002	19	0.002
Responsiveness & Cellular Component	0.4	0.002	19	0.011
TATA box & Structural variables	0.1	0.002	8	0.098
TATA box & Molecular Function	0.3	0.002	26	0.213
TATA box & Biological Process	0.5	0.002	15	0.131
TATA box & Cellular Component	0.3	0.002	12	0.260
Cho et al. – EDA (grouping 1)	0.4	0.001	23	0.318
Cho et al. – EDA (grouping 2)	0.4	0.001	6	0.115
Cho et al. – G&S SHAVING (grouping 1)	0.6	0.002	45	0.006
Cho et al. – G&S SHAVING (grouping 2)	0.6	0.002	36	0.003
Gasch et al. – EDA (grouping 1)	0.4	0.001	17	0.005
Gasch et al. – EDA (grouping 2)	0.4	0.001	21	0.004
Gasch et al. – G&S SHAVING (grouping 1)	0.6	0.001	56	0.023
Gasch et al. – G&S SHAVING (grouping 2)	0.7	0.001	35	0.019

This table shows the CF, Confidence and Support thresholds set in each experiment as well as the total number of rules and the FDR obtained in each case.

**Table 2:** Structural variables

Sup.	Conf.	CF	Association rule
0.12	0.40	0.15	*length *= *LOW *→ *G *+ *C *= *HIGH*
0.12	0.38	0.14	*G *+ *C *= *LOW *→ *length *= *HIGH*
0.12	0.41	0.16	*G *+ *C *= *HIGH *→ *length *= *LOW*
0.12	0.40	0.14	*length *= *HIGH *→ *G *+ *C *= *LOW*
0.13	0.41	0.17	*intLength *= *LOW *→ *intGC *= *LOW*
0.13	0.43	0.18	*intGC *= *LOW *→ *intLength *= *LOW*
0.13	0.44	0.21	*intGC *= *HIGH *→ *intLength *= *HIGH*
0.13	0.44	0.22	*intLength *= *HIGH *→ *intGC *= *HIGH*
0.18	0.63	0.24	*intLeng*. = *HIGH *→ *orient*. = *DIV*
0.23	0.56	0.15	*intLeng*. = *MED *→ *orient*. = *TAN*
0.20	0.40	0.16	*orient*. = *TAN *→ *intGC *= *LOW*
0.20	0.68	0.37	*intGC *= *LOW *→ *orient *= *TAN*
0.19	0.36	0.10	*orient*. = *DIV *→ *intGC *= *HIGH*
0.19	0.65	0.27	*intGC *= *HIGH *→ *orient*. = *DIV*
0.13	0.42	0.17	*intGC *= *LOW *→ *G *+ *C *= *LOW*
0.13	0.41	0.17	*G *+ *C *= *LOW *→ *intGC *= *LOW*
0.14	0.46	0.23	*G *+ *C *= *HIGH *→ *intGC *= *HIGH*
0.14	0.46	0.23	*intGC *= *HIGH *→ *G *+ *C *= *HIGH*
0.038	0.48	0.12	*chr *= 16 → *intLeng*. = *MED*
0.010	0.41	0.17	*chr *= 3 → *gct *= *HIGH*
0.015	0.39	0.14	*chr *= 9 → *intGC *= *HIGH*

This table shows the selected rules involving structural features.

Once the rules are obtained, a global significance value of the rule sets is needed to ensure the quality of those rules. In order to do that we estimated the number of rules that were obtained by chance. For this purpose we generated 100 randomized independent datasets and extracted rules from each of them. The estimated number of false rules was calculated as the mean of the number of rules obtained from each of these 100 randomized datasets. This way, we can calculate a False Discovery Rate (FDR) which allows us to check the quality of the rule sets [44] (see Table 1). Since the FDRs obtained are very low, we can argue that very few rules were generated by chance and that the vast majority of the rules obtained represent real biological associations, proving the significance of the methodology. It is not the aim of this paper to provide a biological interpretation of all of them but to show that significant associations are obtained and that many of them agree with previous results in the field. A deeper biological analysis of the rest of rules will be the topic for future works.

Rules shown in this paper were selected according to expert knowledge and information extracted from the literature. They intend to be understandable statements, either statistically supported by previous work or that could be framed in the light of current knowledge. Complete rule sets are provided on request. Association rules with more than one item in the antecedent/consecuent were also obtained and will be considered in future works. Note that the three GO ontologies were studied separately. For the extraction of rules involving biclusters several groupings were studied. By varying some parameters of the bicluster algorithms we can obtain slightly different sets of biclusters. We selected the best groupings according to the GAP value [6].

#### Structural features

The association rules obtained (Table 2) have captured all of the previously reported relationships between the length and base composition of genes and the upstream intergenic sequences. Indeed, the pioneering description of the yeast genome by Dujon (1996) [19] noted that intergenic spacers between divergently oriented genes are longer and G+C richer than spacers separating tandemly oriented genes.

Rules in Table 2 also express the negative correlation between the length and the G+C content of yeast genes (Spearman's *r *= *-*0.25, *p *< 0.0001) [15]. One may argue that confidence and certainty factor values are low, i.e. ~0.40 and ~0.14 respectively. Nevertheless, these values were expected for these rules: Spearman's correlation obtained by Marin et. al. is equal to *-*0.25, implying that G+C content and ORF length are not independent and that there is some negative correlation between both variables. This is clearly stated by rules in Table 2.

Likewise, rules in Table 2 state the positive correlation between length and G+C for spacers, and also the general compositional correlation between genes and upstream spacers [20].

The biological significance of these relationships is not yet fully understood. The greater length of divergent spacers is certainly related to the presence of two promoters or partially shared promoters. The positive relationship between intergenic length and G+C content is likely to be mediated by the effect of meiotic recombination that occurs predominantly in divergent intergenic spacers and increases G+C content through GC-biased mismatch repair [45-47]. Likewise, the correlation between the G+C content of intergenic spacers and the neighboring genes might arise through a combination of GC-biased mutation during recombination mismatch repair and a selective advantage for greater chromatin openness [46].

#### Protein amount and responsiveness

The final product of the genes considered in this work is a protein. The amount of protein present in the cell, and the ability to adapt it to changing conditions, depend upon the kinetics of complicated processes: transcription and mRNA processing, export from nucleus, mRNA translation and protein turnover (reviewed by Perez-Ortin et al [48]). We have taken advantage of the recent availability of estimates of the protein amount (Ghaemmaghami et al [34]) and of the ability to change the expression level (responsiveness) as an adaptive response to new conditions [35] to search for association rules of protein amount and responsiveness with other genomic variables (Table 3).

**Table 3:** Protein abundance, responsiveness and TATA box

Sup.	Conf.	CF	Association rule
0.092	0.48	0.12	*proteinAbundance *= *HIGH → length *= *MEDIUM*
0.087	0.45	0.22	*proteinAbundance *= *LOW → length *= *HIGH*
0.10	0.40	0.16	*responsiveness *= *HIGH → G *+ *C *= *HIGH*
0.10	0.35	0.13	*G *+ *C *= *HIGH *→ *responsiveness *= *HIGH*
0.11	0.39	0.14	*responsiveness *= *LOW → G *+ *C *= *LOW*
0.074	0.40	0.15	*proteinAbundance *= *HIGH → G *+ *C *= *HIGH*
0.096	0.37	0.12	*responsiveness *= *HIGH → intGC *= *HIGH*
0.11	0.44	0.21	*responsiveness *= *HIGH *→ *intLength *= *HIGH*
0.11	0.38	0.17	*intLength *= *HIGH → responsiveness *= *HIGH*
0.10	0.37	0.10	*responsiveness *= *LOW → intLength *= *LOW*
0.055	0.41	0.17	*TATA *= *yes → intGC *= *HIGH*
0.058	0.44	0.21	*TATA *= *yes → intLength *= *HIGH*

This table shows some rules obtained when looking for relations between the protein abundance, the responsiveness, the TATA box and the rest of variables.

It can be seen that protein abundance is negatively related to gene length. Such result is expected since a number of papers have previously reported a negative correlation between gene length and mRNA levels [15-17,49]. Another rule in Table 3 relates abundant proteins to G+C rich genes, this result corroborates a previous result noting positive correlation between G+C content and transcription level [15]. Likewise, responsiveness appears positively related to the G+C content of the gene, and also to the the length and the G+C content of the upstream spacer. Finally, it is worth noting the association found between the presence of TATA box and the length and G+C content of the upstream spacers.

The above results suggest that, during evolution, yeast protein coding DNA tended to be shortened and to be enriched in G+C content as a response for increasing mRNA concentration and responsiveness.

Shortening of mRNA seems to be due to a selection pressure for reducing the size of abundant proteins to minimize transcriptional and translational costs. Additionally, as the progression of the RNA polymerase through the DNA causes a change in superhelical density [50-53] it is likely that the shorter a gene is, the lower the change in superhelical density generated by transcription. Consequently the changes in supercoiling downstream of the RNA polymerase will be higher in long genes than in short genes. Therefore, a negative effect on the efficiency of progression of the RNA polymerase could occur at the proximal 3'-end regions of long genes.

The relationship between G+C content and gene expression is less intuitive. Since the lower the GC content, the lower the efficiency of transcription, it cannot be argued that a putative more energy-demanding process of opening G+C-rich dsDNA by RNA polymerase vs. low G+C content genes is determining this correlation. Instead, a different chromatin structure of the DNA may determine its efficiency of transcription. The importance of chromatin structure in transcription modulation has been shown in a high-throughput study on transcription of the yeast genome under conditions of depletion of histone H4 [54-56]. In this sense, it has been observed that DNA may have conformational information that determines its capability to interact with DNA topoisomerase I and nucleosomes [57]. Similarly, it has been shown that the nucleosome position is determined by different DNA segments according to the G+C content [58]. In addition, it has been shown that a structural change in an alternating G+C sequence causes both a transcriptional block and a negative supercoiling [59].

These results can be accommodated in the frame of the model for chromatin organization in this genome by Filipski and Mucha [46] (see also references therein), which suggests that the intergenic (G+C-rich) regions between divergently transcribed ORFs would occupy an external position, thus facilitating an open conformation of the chromatin which, in turn, facilitates recombination and greater regulatory possibilities. The greater possibilities for regulation of divergently transcribed genes were unveiled by Cho et al. [32] analyzing the mRNA level fluctuation through the cell cycle. They observed that among the cell-cycle regulated genes (occupying adjacent positions), there is an excess of divergently transcribed (51%) in relation to those that are tandemly (38%) or convergently (11%) transcribed.

#### GO terms

We also obtained association rules linking GO terms. Warringer & Blomberg [49] showed that the gene GO annotations are dependent on the length of the gene. Therefore, it should be captured by our method. We can see that more than 60% of the extracted rules with one item in the antecedent and one in the consequent involving GO terms and structural variables present "ORF length" as a consequent. In addition, Warringer & Blomberg found significant functional overrepresentations for different protein size classes; for example the term "DNA helicase activity" is enriched among the largest proteins (more than 771 amino acids) while the term "cytochrome-c oxidase activity" is enriched among the smallest proteins (less than 202 amino acids). Table 4 shows these features.

**Table 4:** GO terms and structural variables. First approach

Sup.	Conf.	CF	Association rule
0.0041	0.88	0.84	*GO = DNA helicase activity → length *= *HIGH*
0.0017	1	1	*GO = cytochrome-c oxidase activity → length *= *LOW*
0.023	0.57	0.39	*GO = plasma membrane → length *= *HIGH*

This table shows some rules obtained when looking for relations between the GO terms and the structural variables. These rules were obtained with the first approach, i.e. when considering all the rules involving GO terms.

However, lots of rules were obtained involving GO terms, some of them providing almost the same information. These rules can be merged into one more general without loosing relevant information, and thus considerably reducing the number of rules obtained (more details are given in the Methods section). Table 5 shows the number of rules before and after the rule filtering as well as the rule reduction rate for each experiment. The mean rule reduction rate is 38.8%, being 68% the higher value. Hence, the size of many of the rule sets is being reduced to almost a half of their original size. The rule sets before the filtering are provided on request. Although some information might be lost when filtering, it is worth losing this information in order to gain clarity in the final rule sets. Furthermore, if during the analysis of the resultant rule sets there appears a rule which is of special interest, the filtering process can be omitted in order to obtain as much information as possible regarding that relation. Some of the rules obtained after the rule filtering are given in Table 6. For instance, that table shows a rule relating "structural constituent of ribosome" and small proteins matching the title of the work by Godfried et al. [60].

**Table 5:** GO terms. Rule reduction rate

Variables	Number of rules before	Number of rules after	Rule reduction rate
Molecular Function & Structural variables	38	20	47%
Biological Process & Structural variables	11	7	36%
Cellular Component & Structural variables	24	12	50%
Protein abundance & Molecular Function	34	19	44%
Protein abundance & Biological Process	37	21	43%
Protein abundance & Cellular Component	23	14	39%
Responsiveness & Molecular Function	45	23	49%
Responsiveness & Biological Process	28	19	32%
Responsiveness & Cellular Component	50	19	62%
TATA box & Molecular Function	53	26	51%
TATA box & Biological Process	17	15	12%
TATA box & Cellular Component	37	12	68%
Cho et al. – EDA (grouping 1)	24	23	4%
Cho et al. – EDA (grouping 2)	6	6	0%
Cho et al. – G&S SHAVING (grouping 1)	98	45	54%
Cho et al. – G&S SHAVING (grouping 2)	79	36	54%
Gasch et al. – EDA (grouping 1)	21	17	19%
Gasch et al. – EDA (grouping 2)	25	21	16%
Gasch et al. – G&S SHAVING (grouping 1)	95	56	41%
Gasch et al. – G&S SHAVING (grouping 2)	77	35	55%

This table shows the number of rules obtained in the experiments where GO terms are involved before and after applying the rule reduction. There is also a column indicating the rule reduction rate.

**Table 6:** GO terms and structural variables. Second approach

Sup.	Conf.	CF	Association rule
0.028	0.77	0.67	*GO = structural constituent of ribosome → length *= *LOW*
0.01	0.78	0.69	*GO = helicase activity → length *= *HIGH*

This table shows some rules obtained when looking for relations between the GO terms and the structural variables. These rules were obtained with the second approach, i.e. groups of rules representing the same knowledge are merged into one general rule.

#### Gene expression data

In this section we show relations between gene expression patterns and their functional/structural features. The aim of the work is not to provide a biological interpretation for all the gene expression profiles found but to show the ability of the method to unveil interesting biological associations between gene expression profiles and the rest of features in an intuitive and graphic way. Following these ideas we selected six of the biclusters with a clear gene expression profile and which are present in interesting and confident association rules. These selected relations are shown in Table 7.

**Table 7:** Biclusters

Sup.	Conf.	CF	Association rule
0.0029	0.54	0.45	*bicluster *= 1 → *GO *= *non-membrane-bound organelle*
0.0033	0.61	0.45	*bicluster *= 1 → *GO *= *nucleus*
0.0018	0.68	0.46	*bicluster *= 2 *→ length *= *MEDIUM*
0.0022	0.80	0.74	*bicluster *= 2 *→ responsiveness *= *HIGH*
0.0012	0.43	0.40	*bicluster *= 2 → *GO *= *oxidoreductase activity*
0.0039	0.65	0.5	*bicluster *= 3 → *GO *= *nucleus*
0.0029	0.48	0.44	*bicluster *= 3 → *GO *= *DNA metabolism*
0.0033	0.81	0.73	*bicluster *= 4 *→ length *= *LOW*
0.0036	0.89	0.85	*bicluster *= 4 → *G *+ *C *= *HIGH*
0.0037	0.90	0.89	*bicluster *= 4 → *GO *= *non-membrane-bound organelle*
0.0037	0.90	0.89	*bicluster *= 4 *GO *= *biosynthesis*
0.0037	0.90	0.87	*bicluster *= 4 *→ GO *= *protein complex*
0.0035	0.86	0.78	*bicluster *= 4 → *GO *= *organelle part*
0.0035	0.86	0.85	*bicluster *= 4 *→ GO *= *cytosol*
0.0035	0.86	0.85	*bicluster *= 4 → *GO *= *structural molecule activity*
0.0107	0.92	0.89	*bicluster *= 5 → *length *= *HIGH*
0.0073	0.63	0.41	*bicluster *= 5 *responsiveness *= *MEDIUM*
0.0019	0.71	0.69	*bicluster *= 6 *→ chr *= *II*
0.0017	0.64	0.61	*bicluster *= 6 → *GO *= *macromolecule biosynthesis*
0.0017	0.64	0.62	*bicluster *= 6 → *GO *= *cytosol*

This table shows some rules obtained when looking for relations between the gene expression patterns discovered by the biclustering algorithms and the rest of variables.

**Figure 2:**
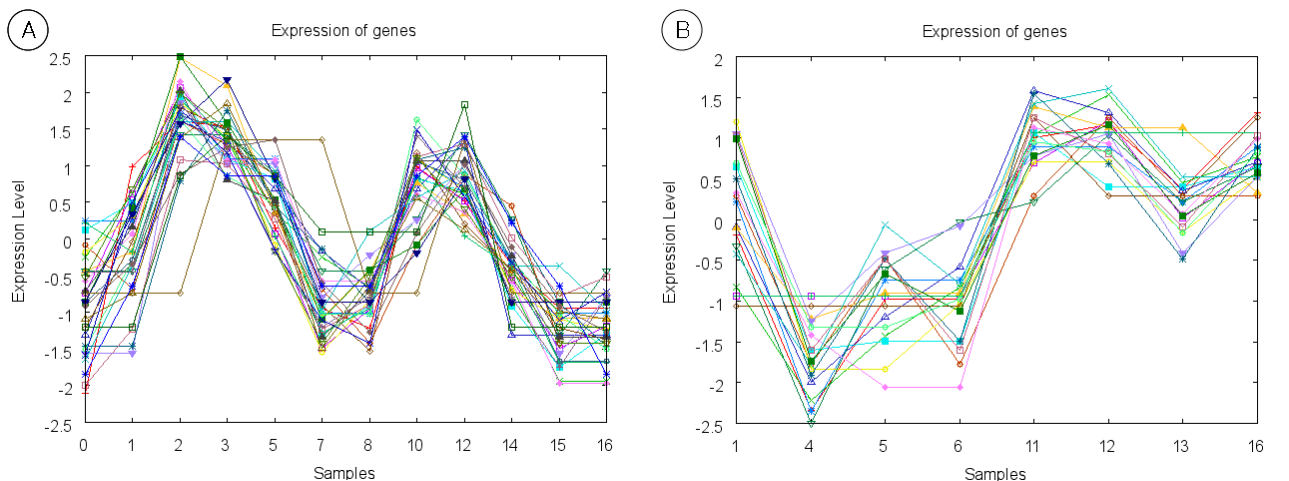
**Biclusters 1 & 2**. This figure shows the gene expression pattern represented by biclusters 1 (A) and 2 (B).

**Figure 3:**
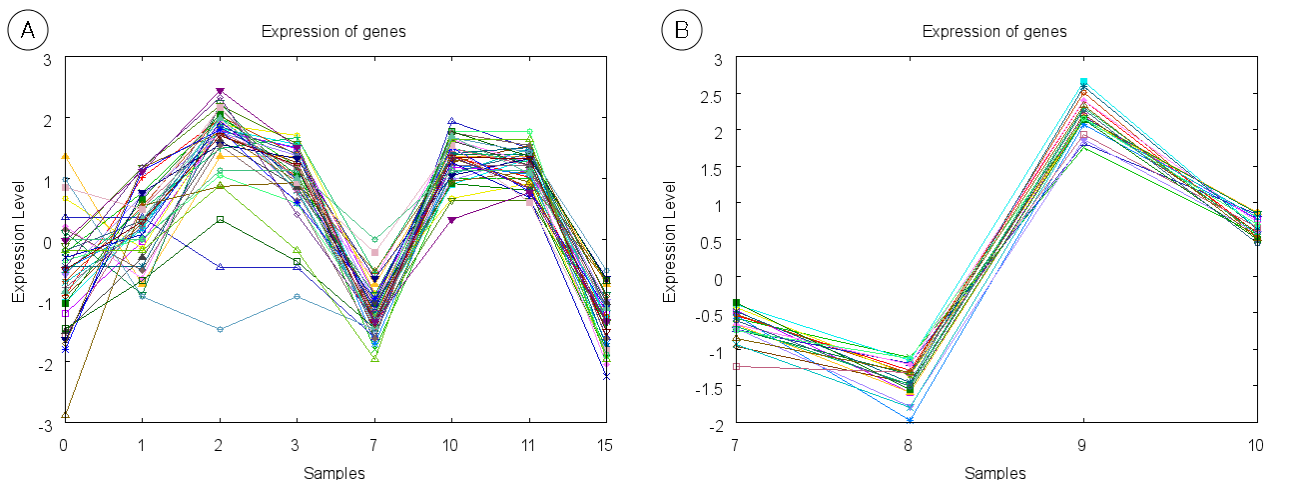
**Biclusters 3 & 4**. This figure shows the gene expression pattern represented by biclusters 3 (A) and 4 (B).

The first four biclusters in Table 7 represent gene expression profiles obtained from the Cell Cycle microarray experiments. Association rules in Table 7 state that bicluster 1 is formed by genes which products are located into the nucleus and in some non-membrane-bound organelles (the definition of non-membrane-bound organelle includes ribosomes, the cytoskeleton and chromosomes). This bicluster was obtained by the EDA biclustering algorithm and Figure 2A depicts the expression pattern it represents. As can be seen, bicluster 1 contains genes over-expressed at the beginning of the cell cycle and under-expressed at the end. It is clear the periodicity of the expression levels of these genes across the two cell cycles comprised in the microarray experiments dataset. Bicluster 2 was also obtained by the EDA biclustering algorithm. ORFs associated to this bicluster have medium length and high responsiveness and carry out an oxidoreductase function. The expression pattern represented by this cluster can be seen in Figure 2B. The next two rules in Table 7 refer to bicluster 3 which was obtained by the EDA biclustering algorithm. ORFs in bicluster 3 yield proteins which carry out their activities into the nucleus and participate in the DNA metabolism. Looking at Figure 3A we can confirm the correspondence between the biological process DNA metabolism and the expression behavior of the genes belonging to the cluster. These genes are over-expressed in the S phase of cell cycle (samples 2–3 and 10–12), in which DNA replication takes place. Finally, some relations are shown for bicluster 4 (Figure 3B). This bicluster was obtained by the Gene & Sample Shaving biclustering algorithm and represents ORFs which gene expression varies sharply from under-expressed to over-expressed when the change of cell cycle takes place (time points 7 to 10). Rules in Table 7 relate bicluster 4 to short ORFs with a high G+C proportion. This makes sense since as was described above it is known that short ORFs tend be GC rich.

**Figure 4:**
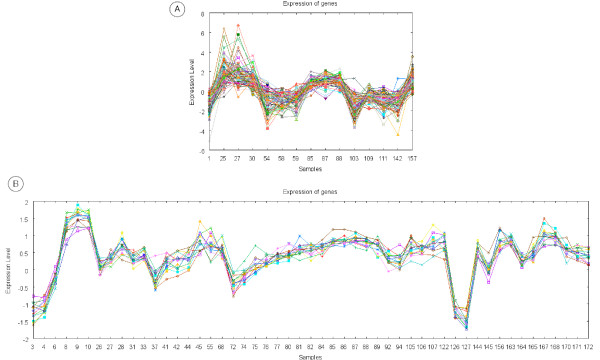
**Biclusters 5 & 6**. This figure shows the gene expression pattern represented by biclusters 5 (A) and 6 (B).

The last 5 rules involve gene expression patterns obtained from the dataset by Gasch et al. Bicluster 5 was obtained by the EDA biclustering algorithm and bicluster 6 by the Gene & Sample Shaving algorithm (see Figures 4A &4B). This dataset is formed by a broad variety of experiments, therefore the obtained biclusters contain columns from very different experiments. For example, bicluster 5 contains columns from 9 different experiment sets. It depicts the gene expression profile of 74 genes under 15 experimental conditions. Genes belonging to this bicluster are large genes which tend to have *MEDIUM *responsiveness. The last three rules involve bicluster 6. This bicluster is specially interesting since it contains many columns (51) and it presents a very clear expression profile. The associations found describe these genes as belonging to chromosome II and being annotated in the terms *macromolecule biosynthesis *and *cytosol*.

#### Fuzzy and crisp results comparison

Differences between the crisp and fuzzy results have been observed. For the extraction of the crisp association rules the continuous domains where divided into three intervals by using the percentiles *p*_33 _and *p*_66_. Two more rule sets were obtained by the fuzzy and the crisp algorithm respectively. The same thresholds were set for both algorithms: 0.004 for Support and 0.5 for Confidence and CF. 22893 rules were obtained with the fuzzy algorithm while 27304 were generated by the crisp algorithm. First of all, the Supports, Confidences and CFs of the rules present in both rule sets were compared. 11655 rules were shared between the two rule sets. In order to determine whether the values of the measures obtained by the fuzzy and the crisp version were significantly different, three ANOVAs were carried out (Table 8). As can be seen, statistically significant differences appear for Supports, Confidences and CFs. Variations in the values of the quality measures were expected due to the way the fuzzy methodology models the borders between adjacent labels. Mean crisp quality values are higher than their fuzzy counterparts, which means that crisp measures tend to be higher than the fuzzy ones. Since fuzzy logic is proved to be a superior technology to enhance the modeling of linguistic concepts and the processing of imprecise data, we can argue that the crisp algorithm tends to provide higher quality values than they really are, thus showing the necessity of using fuzzy techniques.

**Table 8:** ANOVAs for Fuzzy – Crisp comparison

Rule quality measure	*p-value*	Mean-Crisp	Mean-Fuzzy
Support	1, 80*E*-018	0.0080	0.0073
Confidence	1, 13*E*-082	0.777	0.757
Certainty Factor	1, 47*E*-049	0.622	0.606

This table shows the results of the ANOVAs carried out to compare fuzzy and crisp Supports, Confidences and Certainty Factors.

**Figure 5:**
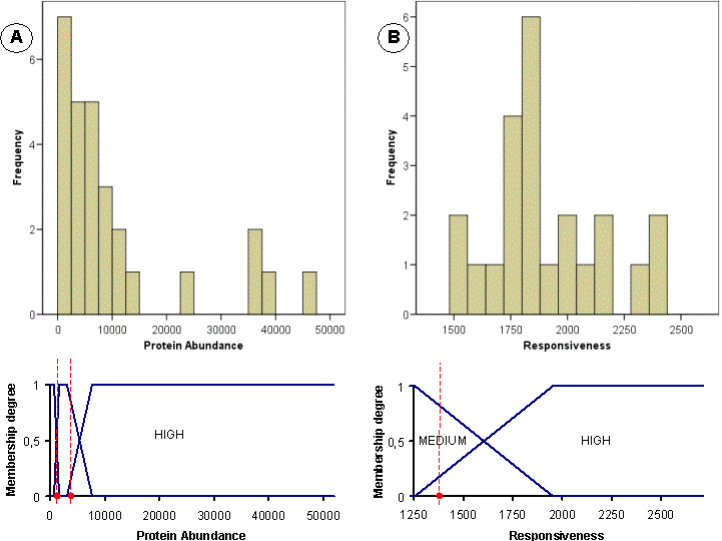
**Comparison between fuzzy and crisp results 1**. A) The histogram shows the distribution of the genes annotated in the term *electron transport *along the protein abundance domain. The graph below describes how the fuzzy sets are defined in this domain. The red dashed lines show the percentiles *p*_33 _and *p*_66_, i.e. the borders of the crisp sets. B) The same but for the genes annotated in the term *snoRNA binding*. Only the percentile *p*_66 _is shown in this case.

**Figure 6:**
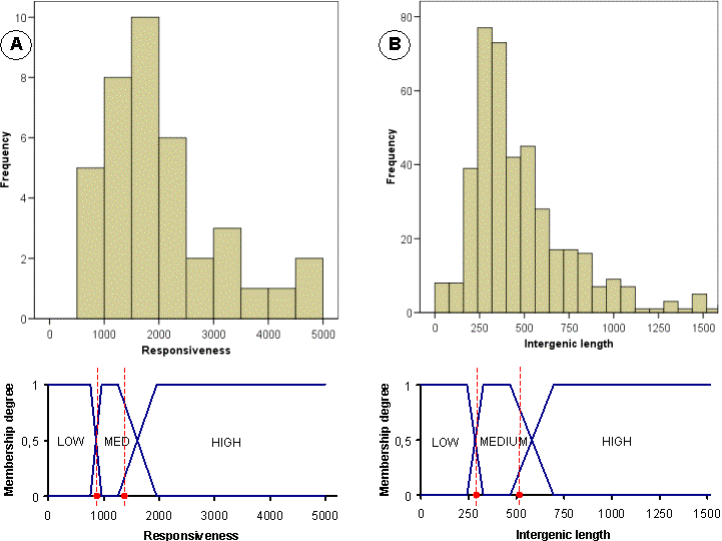
**Comparison between fuzzy and crisp results 2**. A) The histogram shows the distribution of the genes that belong to bicluster 5 along the responsiveness domain. The graph below describes how the fuzzy sets are defined in this domain. The red dashed lines show the percentiles *p*_33 _and *p*_66_, i.e. the borders of the crisp sets. B) The same but for the genes located at chromosome 16 and the intergenic length domain.

Some concrete examples that also show the necessity of using fuzzy methodologies are provided in Table 9. This table shows some rules which quality measures vary significantly between the crisp and fuzzy version. For example, the fuzzy Confidence and fuzzy CF of the first rule in Table 9 are lower than their crisp counterparts. Its fuzzy Support is also lower than the crisp Support. By analyzing Figure 5A it can be understood why the crisp values are lower. This Figure shows how the genes annotated in the term *electron transport *are distributed along the protein abundance domain. It also shows how the linguistic labels are defined in the fuzzy and crisp algorithms. Looking at the histogram in Figure 5A, it can be seen that there appear many genes in the border between the *MEDIUM *and *HIGH *labels. Most of these genes are considered as *LARGE *by the crisp algorithm while they are "a bit" *MEDIUM *and "a bit" *LARGE *in the fuzzy one. This makes the fuzzy algorithm to count fewer *LARGE *genes annotated in *electron transport *and therefore to obtain lower Confidence, CF and Support values. The same reasoning holds for the next two rules in Table 9, their corresponding graphs are shown in Figures 6A and 6B. In the case of the last rule in Table 9, Support, Confidence and CF values are slightly higher in the fuzzy version than in the crisp one. Looking at Figure 6B it can be seen that many of the genes located at the chromosome 16 present values of their intergenic lengths in the borders that divide the *MEDIUM – LOW *and *MEDIUM – HIGH *sets. In this case, the *MEDIUM *fuzzy set not only includes "completely" (i.e. membership degree 1) almost all the genes that are included in the *MEDIUM *crisp set, but also there are many genes that belong to it with a lower membership degree and that are not included in the crisp set. This causes the increase in the fuzzy support value, as the number of genes located at chromosome 16 is the same in the crisp and fuzzy algorithm. Since the number of genes of chromosome 16 with a *MEDIUM *intergenic length is increased, the Confidence and the CF values are also increased.

**Table 9:** Some rules obtained with the fuzzy and crisp algorithms

C-Sup.	F-Sup	C-Conf.	F-Conf	C-CF	F-CF	Association rule
0.0039	0.0030	0.70	0.53	0.60	0.43	*electron transport → proteinAbundance *= *HIGH*
0.0044	0.0036	1	0.81	1	0.75	*snoRNA binding → responsiveness *= *HIGH*
0.0055	0.0044	0.71	0.56	0.58	0.41	*bicluster *= 5 → *responsiveness *= *HIGH*
0.0032	0.038	0.39	0.48	0.09	0.12	*chr *= 16 *→ intLength *= *MEDIUM*

This table shows some rules which were obtained with the fuzzy and crisp algorithms. For each of them their fuzzy and crisp Support, Confidence and CF values are provided.

## Conclusion

We propose a novel fuzzy methodology for the integration and analysis of heterogenous biological data. The main aspect of this fuzzy methodology is a novel fuzzy association rule mining algorithm, the Fuzzy-TD-FP-Growth method.

A dataset based on the yeast genome has been used for the validation of the proposed methodology. The results show interesting associations between structural and functional features of the yeast genome. Many of the obtained biological associations agree with previous works in this field. It demonstrates that by using the proposed methodology significant biological insights can be obtained. It also proves fuzzy association rules to be an intuitive tool to describe biological relations by using linguistic labels and few easy-understandable parameters (support, confidence and certainty factor).

Results also show the importance of using techniques that can model borders in a more realistic way than classical crisp techniques do. The appropriate definition of the concepts introduced in the analysis is crucial since it determines the interpretation that one may obtain from the resultant rule set. In addition, the presence of noise in biological data makes even more necessary to use a fuzzy definition of these concepts.

Future work comprises the development of new rule quality measures and the inclusion of new attributes into the analysis. Furthermore, it will also be interesting to apply the methodology over information obtained from genomes of other species and compare the results.

## Methods

The data table described in section "Dataset" can be easily seen as a transactional database where each gene (i.e. row) represents a transaction and the values in each column form the items of the transaction. The Fuzzy Top-Down Frequent-Parent Growth algorithm is applied over this data table to obtain the fuzzy association rules.

### Fuzzy top-down frequent-parent growth

Experiments showed that a fuzzy version of the *Apriori *algorithm [25,61] cannot deal with our dataset because of the high number of itemsets found, mainly due to the high number of GO terms (data not shown). However, the algorithm method proposed in this work, based on one of the most efficient association rule mining algorithms, the Top-Down Frequent-Parent Growth algorithm, can easily manage this dataset. More about the crisp version of the algorithm can be found in [18].

Initially, the database is scanned in order to get a list of all the *frequent *items, i.e. items with support greater than a threshold. Then, the list is sorted by support in decreasing order. Table 10 shows an example of a frequent item list. Items are then inserted into the Fuzzy-Frequent-Parent tree (FFP-tree) as follows:

**Table 10:** An example of a frequent item list

Index	Item	Support
1	{Gene orientation = TANDEM}	7
2	{Gene length = SHORT}	6
3	{Intergenic length = MEDIUM}	5.98
4	{Intergenic length = LARGE}	4.4
5	{Gene length = LARGE}	4.4
6	{Intergenic length = SHORT}	4
...	...	...

This table shows an example of a frequent item list obtained during the first step of the Fuzzy TD-FP Growth algorithm.

#### Fuzzy-FP tree construction

**Figure 7:**
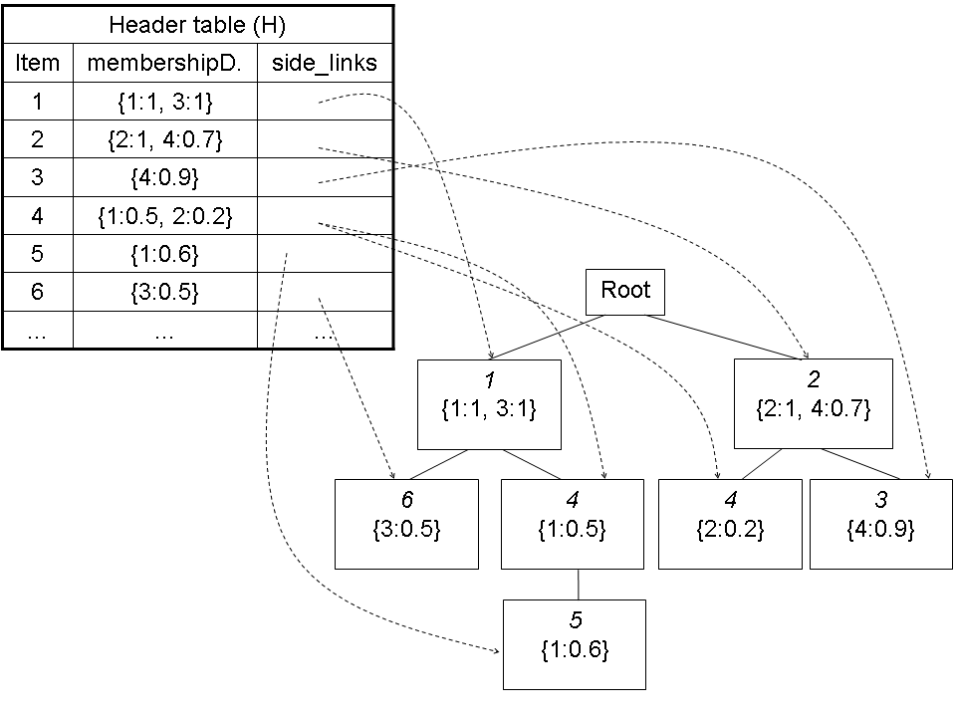
**Complete Fuzzy-FP Tree**. This figure shows an example of a complete Fuzzy-FP tree. Each node contains two membership degree lists, only one is included in the figure for clarity since initially both of them contain the same values.

**Figure 8:**
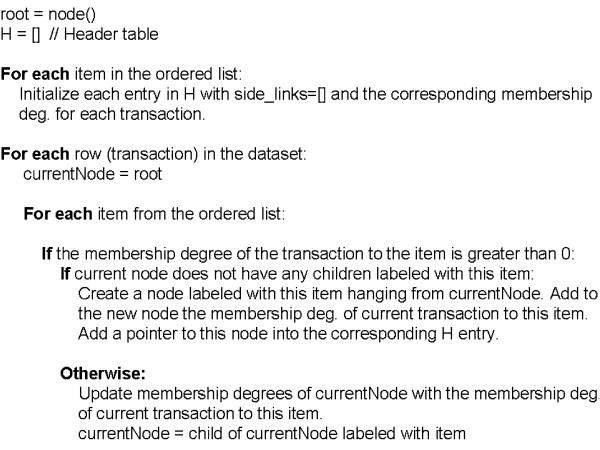
**Procedure for Fuzzy-FP Tree construction**. This figure shows the pseudocode of the algorithm followed to build the Fuzzy-FP tree.

A new scan of the database is carried out. Transactions are considered one by one. The items in each transaction that are present in the list of frequent items are inserted as nodes into the FFP-tree according to their position in the frequent item list. Two transactions share the same upper-path if their first frequent items are the same. All the nodes of each item *I*, are linked by a *side_link*. A vector associated to each node stores the membership degree of the transactions to the corresponding item. For an example in which three transactions are considered see additional file 1: "Fuzzy-Frequent-Parent tree construction". Items are introduced into de FFP-tree according to their position in the sorted frequent item list (Table 10). Next step (frequent itemsets generation) requires the construction of a header table H(Item, membershipDegrees, side_links) that helps to locate the nodes of every item and to compute the fuzzy support of the itemsets. Each entry in H corresponds to an item *I*, and contains the membership degrees of the transactions to *I *and the side_links for this item. A final FFP-tree plus its header table H is shown in Figure 7. Figure 8 describes the procedure for the Fuzzy-FP-tree construction.

#### Frequent itemsets generation

**Figure 9:**
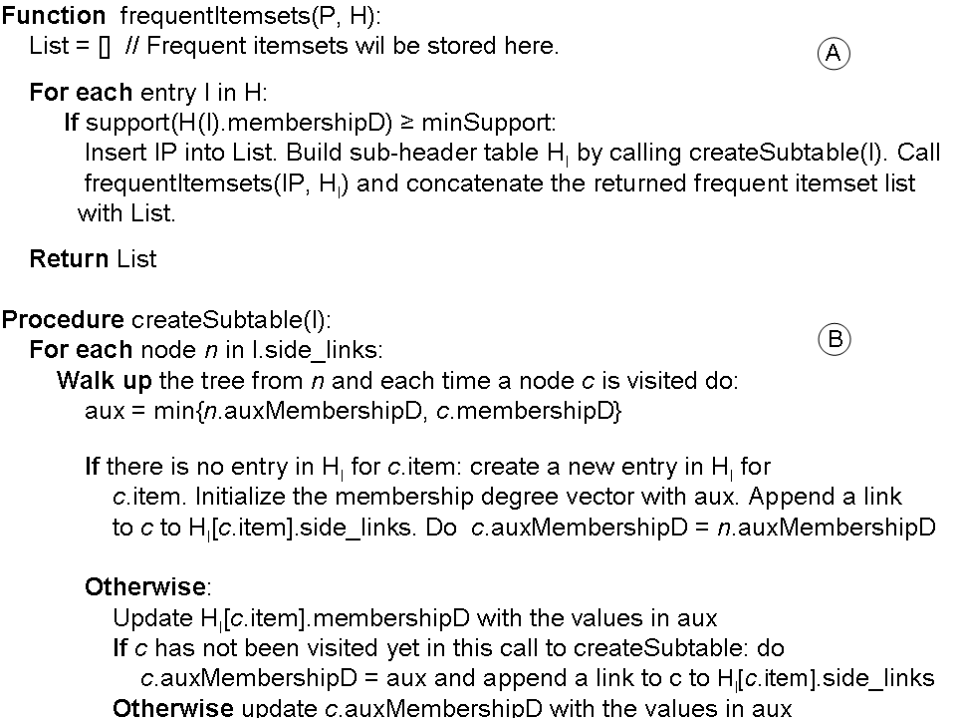
**Frequent itemsets generation**. This figure shows pseudocodes of the algorithm followed to traverse the Fuzzy-FP tree and get the frequent itemsets.

Entries in H are considered one by one. Items in H must be sorted according to their support. For each item *I *in the header table the tree is traversed in a down-top order starting from the nodes labeled with *I*. Nodes labeled with *I *can be reached following the side_links of the corresponding H entry. Every frequent itemset whose last element is *I *is obtained during this walk up. Figure 9 describes this procedure. The reader should note that unlike the crisp version of the TD FP growth algorithm [18] which only needs a counter in each node, in procedure B each node needs an auxiliary membership degree vector that stores the minimum membership degree between the starting node and the current node for their shared transactions. Another key point is that, as in the crisp version, nodes at upper levels are processed before nodes at lower levels. This is crucial because ensures that modifications of these vectors at upper levels do not affect the processing of lower level nodes. For an example of the described procedure to obtain frequent itemsets see additional file 2: "An example of frequent itemsets generation".

Once the list of frequent itemsets has been obtained, association rules which fulfill certain quality measures are generated from each itemset.

### Support, confidence and certainty factors

Fuzzy Support and Confidence are calculated as described in [25]. Given an itemset *I *and a transaction *t*, the membership degree of *t *to the itemset *I *is calculated as min_*i*∈*I *_*μ*_*i*_(*t*), where *μ*_*i*_(*t*) represents the fuzzy membership function which maps the real value of transaction *t *for the attribute in item *i *to the membership grade for the corresponding fuzzy set in *i*.

To avoid some of the drawbacks of the confidence/support framework, *certainty factors *are used [25]. Given an association rule *X *→ *Y*, Equation 1 is employed to calculate the certainty factor value.

(1) $$CF(X\to Y)=\frac{conf(X\to Y)-supp(Y)}{1-supp(Y)},$$

where *conf*(*X *→ *Y*) represents the confidence of the rule, and *supp*(*Y*) represents the support of the consequent. Only association rules with support, confidence and certainty factor greater than the thresholds are generated.

### Rule filtering by using the GO hierarchy

We take advantage of the GO structure in order to filter the rule set. First of all we look for groups of rules involving some GO term and sharing all their items except the GO node. For each group, if there is a GO term in it which is a common ancestor for the rest of GO nodes in this rule set, only the rule involving the common ancestor is maintained and the rest of rules in the group are discarded. This strategy relies on the idea that each Gene Ontology term shares the attributes of all its parents nodes. Since it is ensured that the terms included in the analysis are informative enough by setting an appropriate IC threshold (see Dataset section) the common ancestor represents the most intuitive term. By removing the rest of rules a smaller, clearer and more easily interpretable rule set is provided. For an example of rule filtering see additional file 3: "An example of rule filtering".

## Authors' contributions

FJL helped with the design of the study, implemented the fuzzy association rule mining algorithm, carried out the experiments, helped with the analysis of the results and drafted the paper. AB designed the study and helped to draft the paper. FG helped with the analysis of the results and assisted to draft the paper. CC implemented the bicluster algorithms, obtained the bicluster results and helped to draft the paper. AM helped with the design of the study, analyzed the results and helped to draft the paper. All authors read and approved the final manuscript.

## Supplementary Material

Additional file 1 Fuzzy-frequent-parent tree construction. This file contains an example of how to introduce three transactions into the Fuzzy-Frequent-Parent tree. Click here for file

Additional file 2 An example of frequent itemsets generation. This file contains an example of how to traverse the FFP-tree to obtain the list of frequent itemsets. Click here for file

Additional file 3 An example of rule filtering. This file contains an example in which four rules are merged into only one. Click here for file

## References

[B1] Kanehisa M, Bork P (2003). Bioinformatics in the post-sequence era. Nature Genet.

[B2] Narayanan A, Keedwell EC, Olsson B (2002). Artificial intelligence techniques for bioinformatics. Appl Bioinf.

[B3] Bhaskar H, Hoyle D, Singh S (2005). Machine learning in bioinformatics: A brief survey and recommendations for practitioners. Computers in Biology and Medicine.

[B4] Eisen MB, Spellman PT, Brown P, Botstein D (1998). Cluster analysis and display of genome-wide expression patterns. Proceedings of the Nat Acad Sci USA.

[B5] Tamayo P, Slonim D, Mesirov J, Zhu Q, Kitareewan S, Dmitrovsky E, Lander ES, Golub TR (1999). Interpreting patterns of gene expression with self-organizing maps: methods and application to hematopoietic differentiation. Proceedings of the Nat Acad Sci USA.

[B6] Hastie T, Tibshirani R, Eisen MB, Alizadeh A, Levy R, Staudt L, Chan WC, Botsteinm D, Brown P (2000). Gene shaving as a method for identifying distinct sets of genes with similar expression. Genom Biol.

[B7] Jiang D, Tang C, Zhang A (2004). Cluster analysis for gene expression data: A survey. IEEE Transaction on Knowledge and Data Engineering.

[B8] Rodriguez A, Carazo JM, Trelles-Salazar O (2005). Mining association rules from biological databases. Journal of the American Society for Information Science and Technology.

[B9] Hermert J, Baldock R (2007). Mining Spatial Gene Expression Data for Association Rules. Lecture notes in computer science.

[B10] Dafas PA, d'Avila AS (2007). Discovering Meaningful Rules from Gene Expression Data. Current Bioinformatics.

[B11] Zhong W, Sternberg PW (2007). Automated data integration for developmental biological research. Development.

[B12] Al-Shahrour F, Minguez P, Tarraga J, Medina I, Alloza E, Montaner D, Dopazo J (2007). FatiGO+: a functional profiling tool for genomic data. Integration of functional annotation, regulatory motifs and interaction data with microarray experiments. Nucleic Acids Research.

[B13] Joyce AR, Palsson BO The model organism as a system: integrating "omics" data sets.

[B14] Carmona-Saez P, Chagoyen M, Rodriguez A, Trelles O, Carazo JM, Pascual-Montano A (2006). Integrated analysis of gene expression by association rules discovery. BMC Bioinformatics.

[B15] Marin A, Gallardo M, Kato Y, Shirahige K, Gutierrez G, Ohta K, Aguilera A (2003). Relationship between G+C content, ORF-length and mRNA concentration in Saccharomyces cerevisiae. Yeast.

[B16] Coghlan A, Wolfe KH (2000). Relationship of codon bias to mRNA concentration and protein length in Saccharomyces cerevisiae. Yeast.

[B17] Jansen R, Gerstein M (2000). Analysis of the yeast transcriptome with structural and functional categories: characterizing highly expressed proteins. Nucleic Acids Res.

[B18] Wang K, Tang L, Han J, Liu J (2002). Top down FP-Growth for association rule mining. Proceedings of the 6th Pacific Area Conference on Knowledge Discovery and Data Mining: Taipei, Taiwan.

[B19] Dujon B (1996). The yeast genome project: what did we learn?. Trends Genet.

[B20] Marin A, Wang M, Gutierrez G (2004). Short-range compositional correlation in the yeast genome depends on transcriptional orientation. Gene.

[B21] Agrawal R, Imielinski T, Swami A (1993). Mining association rules between sets of items in large databases. Proceedings Of the ACM SIGMOD INTL Conf on Management of Data (ACM SIGMOD 93): Washington, USA.

[B22] Zadeh LA (1965). Fuzzy sets. Information and Control.

[B23] Zimmerman HJ (2001). Fuzzy sets theory and its applications.

[B24] Delgado M, Marin N, Martin-Bautista MJ, Sanchez D, Vila MA (2003). Mining fuzzy association rules: an overview. Proceedings Of the BISC International Workshop on Soft Computing for Internet and Bioinformatics.

[B25] Delgado M, Marin N, Sanchez D, Vila MA (2003). Fuzzy association rules: General model and applications. IEEE Trans Fuzzy Systems.

[B26] Goffeau A (1997). The yeast genome directory. Nature.

[B27] Castrillo JI, Oliver SG (1996). Yeast as a Touchstone in Post-genomic Research: Strategies for Integrative Analysis in Functional Genomics. J Biochem Mol Biol.

[B28] Wohlschlegel JA, Yates JR (2003). Where's Waldo in yeast?. Nature.

[B29] Dwight SS, Harris MA, Dolinski K, Ball CA, Binkley G, Christie KR, Fisk DG, Issel-Tarver L, Schroeder M, Sherlock G, Sethuraman A, Weng S, Botstein D, Cherry JM (2002). Saccharomyces Genome Database (SGD) provides secondary gene annotation using the Gene Ontology (GO). Nucleic Acids Res.

[B30] The Saccharomyces Genome Database. http://www.yeastgenome.org.

[B31] The Comprehensive Yeast Genome Database. http://mips.gsf.de/genre/proj/yeast.

[B32] Cho R, Campbell M, Winzeler E, Steinmetz L, Conway A, Wodicka L, Wolfsberg T, Gabrielian A, Landsman D, Lockhart D (1998). A genome-wide transcriptional analysis of the mitotic cell cycle. Mol Cell.

[B33] Gasch AP, Spellman PT, Kao CM, Carmel-Harel O, Eisen MB, Storz G, Botstein D, Brown PO (2000). Genomic expression programs in the response of yeast cells to environmental changes. Mol Biol Cell.

[B34] Huh W, Falvo JV, Gerke LC, Carroll AS, Howson RW, Weissman JS, O""'Shea EK (2003). Global analysis of protein localization in budding yeast. Nature.

[B35] Tirosh I, Weinberger A, Carmi M, Barkai N (2006). A genetic signature for inter-species variations in gene expression. Nature Genetics.

[B36] Resnik P (1995). Using information content to evaluate semantic similarity in a taxonomy. Proceedings of the 14th International Joint Conference on Artificial Intelligence, IJCAI: Montreal, Canada.

[B37] The Gene Ontology. http://www.geneontology.org.

[B38] Dubitzky W, Granzow M, Downes C, Berrar D, Berrar DP, Dubitzky W, Granzow M (2004). Introduction to Microarray Data Analysis. A Practical Approach to Microarray Data Analysis.

[B39] Creighton C, Hanash S (2003). Mining gene expression databases for association rules. Bioinformatics.

[B40] Lopez FJ, Blanco A, Garcia F, Marin A (2007). Extracting biological knowledge by fuzzy association rule mining. Proceedings of the IEEE International Conference on Fuzzy Systems: London, UK.

[B41] Madeira S, Olivera A (2004). Biclustering algorithms for biological data analysis: a survey. IEEE/ACM Transactions on Computational Biology and Bioinformatics.

[B42] Preli A, Bleuler S, Zimmermann P, Wille A, Buhlmann P, Gruissem W, Hennig L, Thiele, Zitzler E (2006). A systematic comparison and evaluation of biclustering methods for gene expression data. Bioinformatics.

[B43] Swift S, Tucker A, Vinciotti V, Martin N, Orengo C, Liu X, Kellam P (2004). Consensus Clustering and Functional Interpretation of Gene-Expression Data. Genome Biology.

[B44] H Zhang BP (2004). Using Randomization to Determine a False Discovery Rate for Rule Discovery. Proceedings of the Fourteenth Workshop On Information Technologies And Systems.

[B45] Birdsell JA (2002). Integrating genomics, bioinformatics, and classical genetics to study the effects of recombination on genome evolution. Mol Biol Evol.

[B46] Filipski J, Mucha M (2002). Structure, function and DNA composition of Saccharomyces cerevisiae chromatin loops. Gene.

[B47] Gerton JL, DeRisi J, Shroff R, Lichten M, Brown PO, Petes TD (2000). Global mapping of meiotic recombination hotspots and coldspots in the yeast Saccharomyces cerevisiae. Proceedings of the Natl Acad Sci USA.

[B48] Perez-Ortin JE, Alepuz PM, Moreno J (2007). Genomics and gene transcription kinetics in yeast. Trends Genet.

[B49] Warringer J, Blomberg A (2006). Evolutionary constraints on yeast protein size. BMC Evol Biol.

[B50] Osborne BI, Guarente L (1988). Transcription by RNA polymerase II induces changes of DNA topology in yeast. Genes Dev.

[B51] Brill SJ, Sternglanz R (1988). Transcription-dependent DNA supercoiling in yeast DNA topoisomerase mutants. Cell.

[B52] Pederson DS, Morse RH (1990). Effect of transcription of yeast chromatin on DNA topology in vivo. EMBO Journal.

[B53] Lee MS, Garrard WT (1991). Positive DNA supercoiling generates a chromatin conformation characteristic of highly active genes. Proceedings of the Natl Acad Sci USA.

[B54] Wyrick JJ, Holstege FCP, Jennings EG, Causton HC, Shore D, Grunstein M, Lander ES, Young RA (1999). Chromosomal landscape of nucleosome-dependent gene expression and silencing in yeast. Nature.

[B55] Lee TI, Young RA (2000). Transcription of eukaryotic proteincoding genes. Ann Rev Genet.

[B56] Wyrick JJ, Young RA (2002). Deciphering gene expression regulatory networks. Curr Opin Genet Dev.

[B57] Caserta M, Camilloni G, Venditti S, Venditti P, Di Mauro E (1994). Conformational information in DNA: its role in the interaction with DNA topoisomerase I and nucleosomes. J Cell Biochem.

[B58] Wang YH, Griffith JD (1996). The [(G/C)3NN]n motif: a common DNA repeat that excludes nucleosomes. Proceedings of the Natl Acad Sci USA.

[B59] Peck LJ, Wang JC (1985). Transcriptional block caused by a negative supercoiling induced structural change in an alternating CG sequence. Cell.

[B60] Bollen GHPM, Mager WH, Jenneskens LW, Planta RJ (1980). Small-Size mRNAs Code for Ribosomal Proteins in Yeast. Eur J Biochem.

[B61] Srikant R, Agrawal R (1994). Fast algorithms for mining association rules. Proceedings of the 20th Int'l Conference on Very Large Databases: Santiago, Chile.

