# Supplementary material for: Fuzzy association rules for biological data analysis: A case study on yeast
Source: BMC Bioinformatics. 2008 Feb 19;9:107. doi: 10.1186/1471-2105-9-107 (PMC2277399; doi:10.1186/1471-2105-9-107)
Supplement: Additional file 1 — Fuzzy-frequent-parent tree construction. This file contains an example of how to introduce three transactions into the Fuzzy-Frequent-Parent tree. [file 1471-2105-9-107-S1.pdf]

Fuzzy Frequent Parent Tree construction

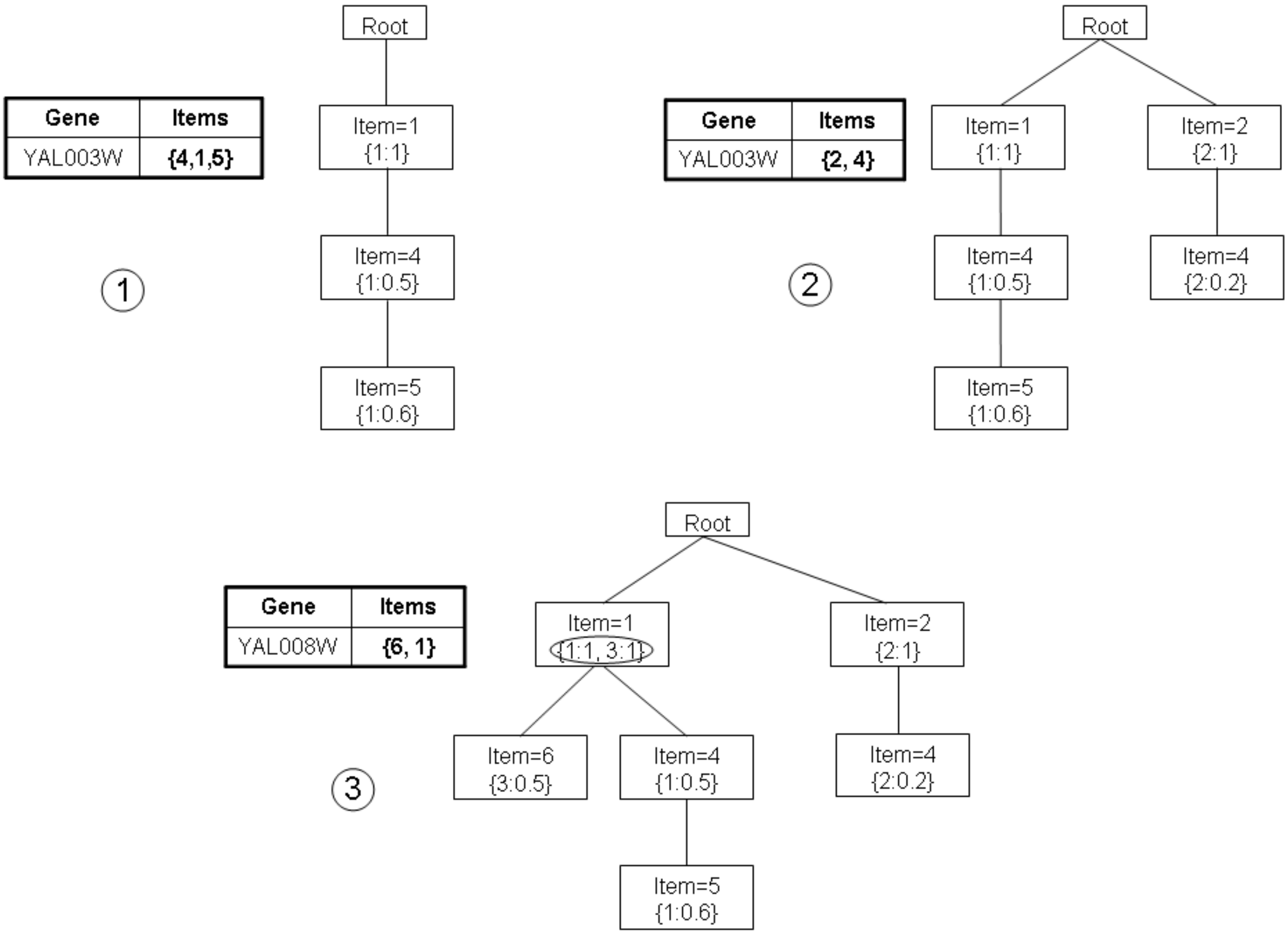

This figure shows graphically the procedure to build a Fuzzy FP Tree. The figure shows the fuzzy transaction introduced in each step beside each tree. Lists under the item number in each node indicate the membership degree of the transactions for the corresponding items. For example, transactions 1 and 3 belong with degree 1 to item 1. Each node contains two membership degree lists, only one is included in the figure for clarity since initially both of them contain the same values.
