# Supplementary material for: Fuzzy association rules for biological data analysis: A case study on yeast
Source: BMC Bioinformatics. 2008 Feb 19;9:107. doi: 10.1186/1471-2105-9-107 (PMC2277399; doi:10.1186/1471-2105-9-107)
Supplement: Additional file 2 — An example of frequent itemsets generation. This file contains an example of how to traverse the FFP-tree to obtain the list of frequent itemsets. [file 1471-2105-9-107-S2.pdf]

An example of frequent itemsets generation

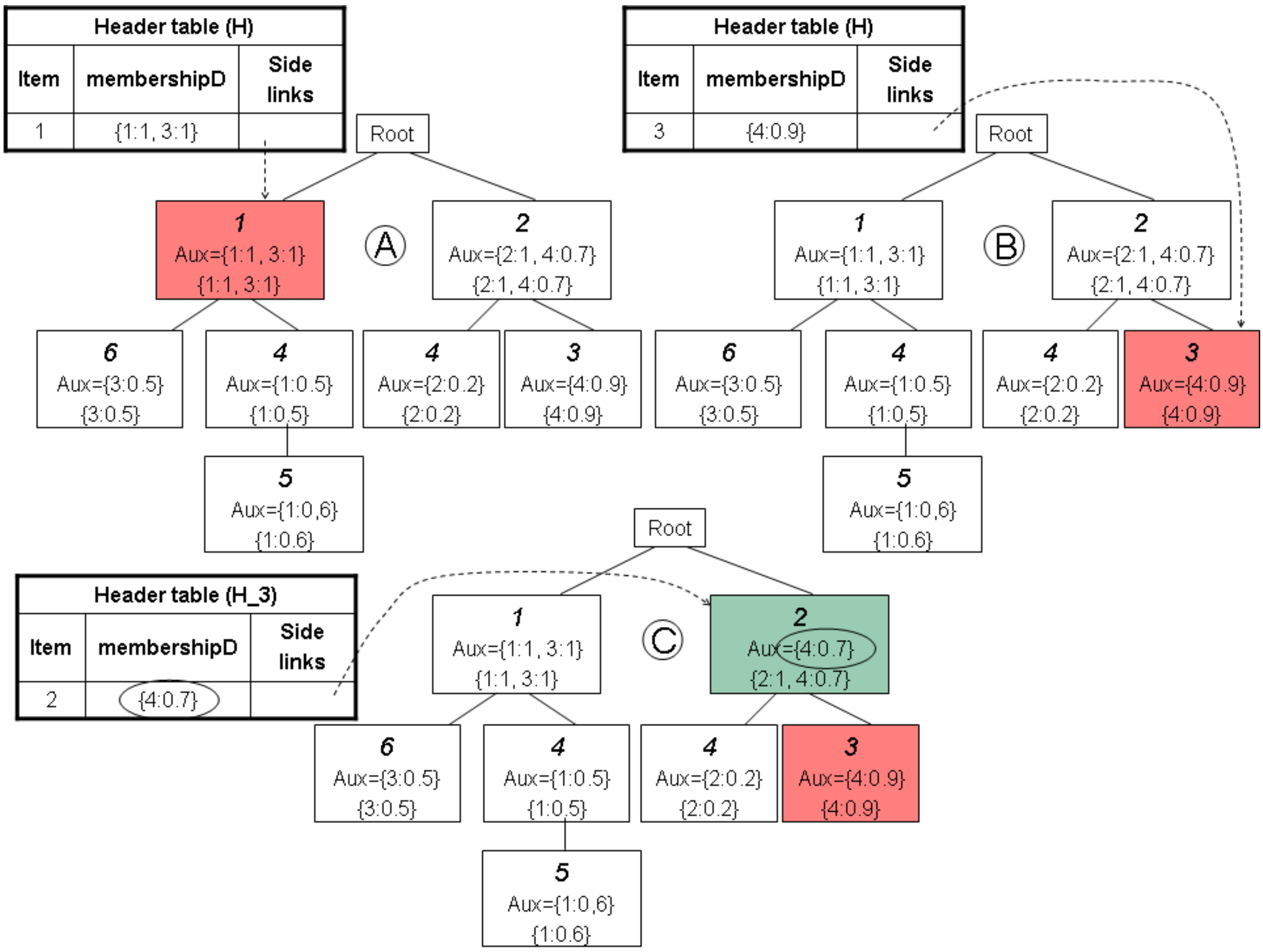

This figure shows an example of the described procedure to obtain frequent itemsets, assuming that the Fuzzy-FP tree was already constructed and it is that of Figure 9.

Firstly, nodes corresponding to item 1 are considered. There is only one node for this item, the colored one in the A tree. The algorithm proceeds to build sub-header table H_1 starting at this node. The root is reached at the first step, so no sub-header table needs finally to be built. Next item in table H is item 2. The situation is the same as for item 1, therefore no sub-header table needs to be constructed. Next item to be considered is item 3. The node for this item is the colored one in the B tree. In the first step of the walk up a node of item 2 is visited. For the sake of clarity, let us suppose that the itemset {2, 3} is frequent. Therefore, the itemset {2, 3} is inserted into frequent itemset list and a new entry for item 2 is added to sub-header table H_3. Membership degrees for the new entry are initialized as shown in Figure C and a pointer to the visited node is added to the side_links vector. The algorithm then proceeds to process the sub-header table H_3 (Figure C). The root is reached at the first step and therefore sub-header table H_3_2 does not need to be built. The algorithm continues considering the next entry in table H and so on. The procedure finishes when the header table and every sub-header table have been processed.
