# Supplementary material for: Fuzzy association rules for biological data analysis: A case study on yeast
Source: BMC Bioinformatics. 2008 Feb 19;9:107. doi: 10.1186/1471-2105-9-107 (PMC2277399; doi:10.1186/1471-2105-9-107)
Supplement: Additional file 3 — An example of rule filtering. This file contains an example in which four rules are merged into only one. [file 1471-2105-9-107-S3.pdf]

An example of rule filtering

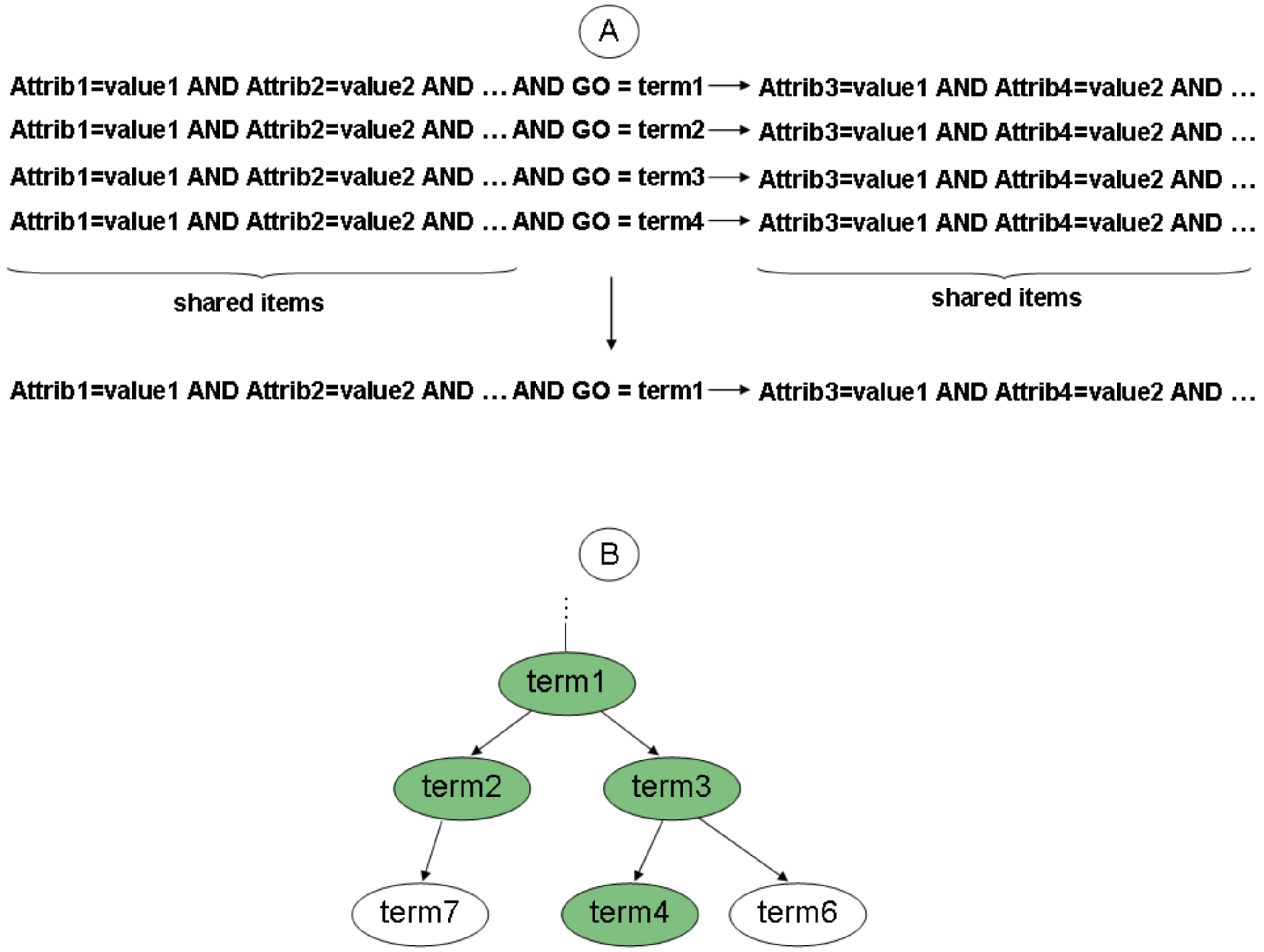

This figure shows an example in which four rules are merged into only one. Part A shows a group of 4 rules sharing all their items except the one involving the GO term. These 4 rules are merged into the more general one. Part B shows the distribution of the terms in the ontology.
